# Bilateral Nephrectomy for Adult Polycystic Kidney Disease Does Not Affect the Graft Function of Transplant Patients and Does Not Result in Sensitisation

**DOI:** 10.1155/2019/7423158

**Published:** 2019-06-11

**Authors:** Maria Irene Bellini, Sotiris Charalmpidis, Paul Brookes, Peter Hill, Frank J. M. F. Dor, Vassilios Papalois

**Affiliations:** ^1^Renal and Transplant Directorate, Hammersmith Hospital, Imperial College Healthcare NHS Trust, London, UK; ^2^Department of Pathology, Hammersmith Hospital, Imperial College Healthcare NHS Trust, London, UK; ^3^Department of Surgery and Cancer, Imperial College, London, UK

## Abstract

**Background:**

Native nephrectomy in Adult Polycystic Kidney Disease (ADPKD) patients is a major operation with controversy related to timing and indications. We present our single centre experience in transplanted patients and future candidates for transplantation.

**Methods:**

Retrospective analysis from an anonymised database of bilateral nephrectomies for ADPKD patients. Results were reported as median, range, and percentage. Differences between groups were tested using ANOVA and t-test. Surgery was performed between January 2012 and July 2018.

**Results:**

Thirty-three patients underwent bilateral native nephrectomy for APKD. 18 had a functioning kidney transplant (transplant group, 55%) while 15 patients were on dialysis (dialysis group, 45%) at the time of surgery; 8 patients of the latter group (24% of the whole cohort) were eventually transplanted. 53% were males, with median age of 55 years (27-71). Indications to surgery were the following: space (symptoms related to the size of the native kidneys or need to create space for transplantation) (59%), recurrent cyst infection (36%), haematuria (15%), pain (24%), and weight loss associated with cystic alteration on imaging (3%). In the transplant group, postoperative kidney function was not affected; haemoglobin serum levels significantly dropped in the whole cohort: 121 (82-150) g/L, versus 108 (58-154) g/L (p<0.001), with 14 patients being transfused perioperatively. Elevation of anti-HLA antibodies was noted in one female patient on dialysis, with no change in DSA levels and no rejection after transplant for all 26 transplanted patients. Median postoperative length of hospital stay was 9 days (6-71). One patient died (3%) after six months. Median follow-up for the whole cohort was 282 days (13-1834). Histopathological examination revealed incidental renal neoplasms in five cases (15%): 1 pT1a papillary renal cell carcinoma and 4 papillary adenomas.

**Conclusions:**

Native nephrectomy for ADPKD could be safely performed in case of refractory symptoms, suspect of cancer or to create space for transplantation. It does not affect graft function or DSA status of transplanted patients or the prospect of transplantation of those on the waiting list.

## 1. Introduction

Autosomal dominant polycystic kidney disease (ADPKD) is the consequence of a heterozygous mutation in one of two genes: PKD1 on chromosome 16 [1], in 80-85% of cases, or PKD2 on chromosome 4 [2]. The mutation leads to dysfunction of the corresponding protein products, polycystin 1 and 2, resulting in aberrant cellular signalling pathways with increased or disorganised cell growth and fluid secretion with fluid accumulation and cyst formation [3, 4].

Clinical manifestations in ADPKD patients include urine-concentration defect, haematuria [3, 5], cyst infection, urinary tract infection [6], loin or abdominal pain, abdominal fullness and discomfort, nephrolithiasis, and hypertension [7]. An association with other systemic manifestations is also frequent: polycystic liver disease, mitral valve prolapse or ventricular hypertrophy [8], intracranial aneurysm, abdominal aortic aneurysm [7], diverticular disease [5], and bronchiectasis [8]. For abdominal aortic aneurysm in particular, they tend to expand more rapidly in transplanted patients, and in the case of massively enlarged polycystic kidneys, the surgical access for a ruptured abdominal aortic aneurysm could be very challenging [9]; therefore variation in clinical symptoms has to be carefully assessed.

The clinical course is variable, suggesting that other genes and environmental factors may play a role [10]. Progression of ADPKD is ultimately defined by the onset of end stage renal failure; ADPKD is the commonest inherited kidney disease and is the fourth commonest cause of kidney failure worldwide [11, 12].

The timing of surgical intervention is controversial for patients with refractory symptoms, especially if they are not in renal replacement therapy. Native nephrectomy of ADPKD is generally performed for recurrent infection, haematuria, space, chronic pain, and tumour suspicion on imaging [13–15]. Literature reports that approximately 20-30% of the whole ADPKD population require surgical intervention [3, 16, 17], although there is an associated morbidity that might jeopardise the possibility for the patients to get transplanted, so routine pretransplant nephrectomy is no longer recommended.

The aim of this study is to review our single centre experience with native bilateral nephrectomy for ADPKD in patients who were waitlisted for, or previously underwent kidney transplant, focusing on timing of surgery, surgical outcomes, postnephrectomy kidney graft function, and sensitisation.

## 2. Patients and Methods

The study was designed as a single-centre retrospective cohort analysis. We identified the patients who have undergone native bilateral nephrectomy of their polycystic kidneys in the period between January 2012 and July 2018 from our prospectively maintained database. All the patients underwent native bilateral nephrectomy through an open midline laparotomy and the specimens were submitted for routine histological evaluation. The decision to proceed with surgery depended on the indications for surgery and the perceived benefits, and it was made following consensus of a multidisciplinary team of surgeons, nephrologists, and radiologists. Based on our protocol, for predialysis patients, we wait until they need dialysis and we perform the nephrectomies after they are established on dialysis for a period of 3 months.

The study was conducted in accordance with institutional ethics regulations; since it was a retrospective chart analysis, no informed consent was required. Demographic, clinical, and laboratory information was extracted in an anonymised way from charts and electronic records, including operation reports and pre- and postoperative clinic notes: indications for surgery, imaging, estimated Glomerular Filtration Rate (eGFR) according to the Modification of Diet in Renal Disease formula [18], Donor Specific Antibodies (DSA), and anti-HLA (Human Leucocyte Antigens), haemoglobin levels before and after surgery, and pathology reports. Patients were divided according to their transplant status: having a functioning kidney transplant (transplant group) or being on dialysis (dialysis group) at the time of native bilateral nephrectomy. Surgical morbidity was categorised according to Clavien classification [19].

Results are reported as median, range, and percentage. Differences between groups were tested using ANOVA and t-test. A critical* p* value of 0.05 was set for statistical significance. Statistical analyses were performed using SPSS v.20.0 (IBM SPSS Statistics for Windows, Version 20.0; IBM Corp, Armonk, NY).

The work has been reported in line with the STROCSS criteria [20].

## 3. Results

Thirty-three consecutive patients were identified during the study period. Of these, 18 had a functioning kidney transplant at the time of the native bilateral nephrectomy (55%)/transplant group, while 15 patients were on dialysis at the time of the operation (45%)/dialysis group; 8 of the patients on dialysis at the time of the operation were later transplanted (24% of the whole cohort). Immunosuppression therapy consisted of alemtuzumab induction and tacrolimus maintenance monotherapy, with early withdrawal of steroids in the first week after transplant. The median follow-up was 282 days (33-1834). One patient (3%) died 6 months after bilateral nephrectomy. 55% of the whole cohort were males (n=18), with median age 55 years (range 27-71), Table 1. Age and sex distributions were the same between the transplant and dialysis groups.

Indications for native nephrectomy were categorised into space (symptoms related to the size of the native polycystic kidneys or need to create space for transplantation) (n=20, 59%), recurrent cyst infection (n=12, 36%), haematuria (n=5, 15%), pain (n=8, 24%), and weight loss associated with cystic alteration on imaging (n=1, 3%), with haemorrhagic and complex cyst content. Indications for surgery did not differ statistically between the transplant and dialysis groups.

**Figure 1:**
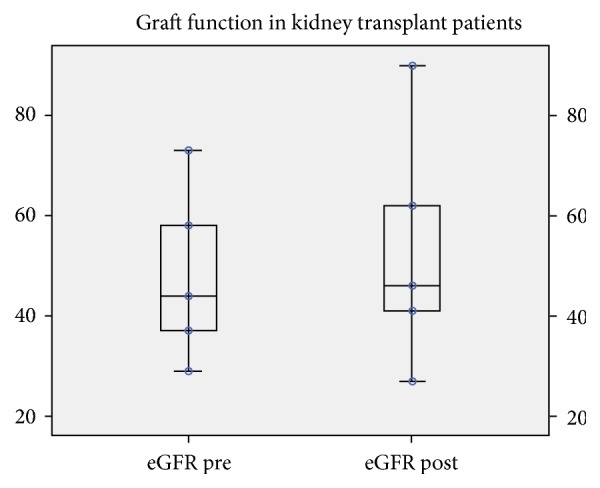
Median eGFR before nephrectomy and on the 1^st^ postnephrectomy day. No significant effect in kidney function was noted (p=0.63). eGFR is reported in ml/min/1.73m^2^.

In the transplant group, median eGFR before and at day 1 after native bilateral nephrectomy did not differ significantly: 44 (29-73), versus 45 (27-90) ml/min/1.73m^2^ (paired t-test, p=0.63), as also shown in Figure 1.

**Figure 2:**
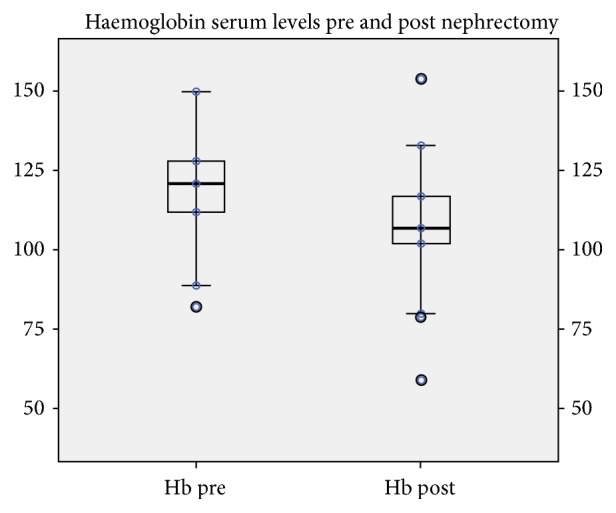
Haemoglobin levels (Hb) before nephrectomy and on the 1^st^ postnephrectomy day, with lower levels postoperatively (p<0.001). Hb is reported in g/L.

Figure 2 shows the significant post-native bilateral nephrectomy change in the haemoglobin serum levels for the whole cohort, 121 (82-150) pre- versus 107 (58-154) g/L postoperatively (p<0.001).

Fourteen patients were transfused perioperatively (Clavien grade II). Elevation of anti-HLA post- compared to pre-bilateral native nephrectomy was noted only in one female dialysis patient, with previous history of pregnancies. For all 26 transplanted patients (18 transplanted before and 8 transplanted after native bilateral nephrectomy), during the follow-up period of this study, DSA levels did not change and there was no rejection episode.

Median postoperative length of hospital stay (LOS) was 9 days (6-71): 56% up to 9 days, 75% up to 11 days, and 97% up to 24 days. One patient developed line sepsis, requiring in-hospital antibiotic treatment during dialysis, and was discharged on day 71. There was no statistical difference between the transplant and the dialysis groups (p=0.28). Two patients developed prolonged ileus that spontaneously resolved within 1 week of the operation (6%) (Clavien grade I); two patients developed abdominal collections and were treated with intravenous antibiotics (Clavien grade II), plus interventional radiology drain insertion in one case (Clavien grade III). One patient developed refractory ascites due to liver disease, finally leading to hepatorenal syndrome (Clavien grade IV); this patient was initially discharged but died 6 months after the operation. No patient underwent relaparotomy postoperatively.

**Figure 3:**
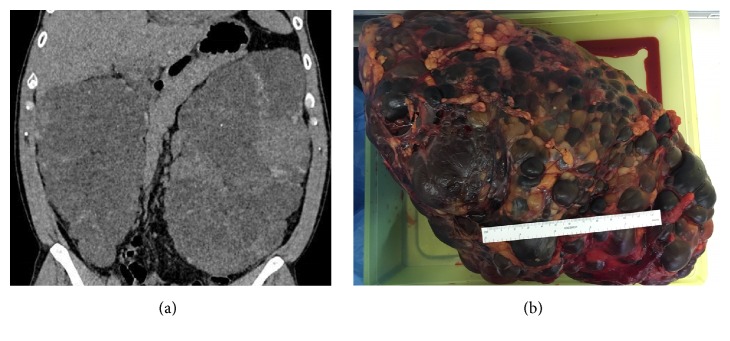
Massive polycystic kidneys leading to an indication for creating space to facilitate transplantation.

The specimens demonstrated typical features of ADPKD in all cases, with replacement of kidney tissue by numerous cysts. Median cyst diameter was 7.5 cm (3.5-10), median kidney length 22 cm (7.5-38), median width 14 cm (6-19), median height 10 cm (3-14), and median weight 1631 g (566-5840). Figures 3(a) and 3(b) demonstrate massive polycystic kidneys leading to an indication for creating space in a patient who underwent transplantation 5 months after the native bilateral nephrectomy.

An association with polycystic liver disease was found in 18 cases, p<0.001 (independent t-test). Histopathological examination revealed incidental renal neoplasms in five cases (15%): 1 pT1a papillary renal cell carcinoma type 1 measuring 4 mm and 4 papillary adenomas.  Table 2 represents incidental pathological findings in correlation to nephrectomy indication and timing of transplantation. The patient with carcinoma did not require further oncological treatment and is well with no evidence of disease recurrence at 18 months of follow-up. None of those neoplasms were evident in prenephrectomy CTs.

## 4. Discussion

ADPKD patients may get massively enlarged kidneys with resultant problems, where surgery is offered to those approaching ESRD or already in renal replacement therapy. Native bilateral nephrectomy is indicated for patients with large kidneys causing pressure symptoms, pain, infection, bleeding, hypertension, and suspicion of malignancy; this operation is also indicated to create space for a renal allograft [21]. The patients who were transplanted prior to the native bilateral nephrectomy did not experience eGFR worsening postoperatively. The comparison of the preoperative e-GFR was made with the one in the immediate postoperative period since this was the timing of the anticipated maximum effect of the stress of the operation and acute kidney graft injury. There were neither episodes of rejection nor elevations of DSA level for all 26 transplanted patients within the follow-up period, indicating that there is no immunological risk related to the native bilateral nephrectomy and this was despite the fact that 14 out of the 33 patients required blood transfusion in the postoperative period. These findings also align with previous studies that recommended nephrectomy for polycystic kidneys only after the transplant [22].

In our cohort, the main indication to native bilateral nephrectomy was space (59%) in the view of a prospective transplant, or because of compression/abdominal fullness. Recurrent cyst infection was also common (36%); particular attention has to be given to frail dialysis patients who could develop sepsis more frequently than the general population [23], or even more to the immunosuppressed transplanted patients. In this latter group, there is also a general alert towards the development of malignancy after transplantation, now becoming the leading cause of death with a functioning graft, due to the effect of immunosuppression [24]. In addition to the immunosuppression risk, the role of ADPKD as a risk factor for renal cell carcinoma is well established; in fact a recent review by Yu et al. highlights the association of polycystic kidney disease with the risk of liver, colon, and kidney cancer [25]. Our centre policy is to monitor carefully any cystic change at imaging in ADPKD transplanted patients; of note, there is no evidence that there is an advantage in preventing malignancy development in APKD by avoiding the use of calcineurin inhibitors, as immunosuppression alone cannot be accounted as responsible for renal cancer in transplanted patients [21, 26].

In the present series, we found five incidental tumours (15%), of which one was a papillary renal cell carcinoma and the other four were benign adenomas. None of those neoplasms were evident in prenephrectomy CTs. The concept of papillary adenoma is controversial and in theory related to a proliferation in tubulopapillary epithelium that has no metastatic potential. Nevertheless, they are very similar histomorphologically to papillary RCCs [27]. It has been suggested that as the size of papillary adenomas increases so does the amount of chromosomal alteration and the potential to transform to cancer. This raises the possibility that papillary adenomas and papillary RCCs represent a continuum of the same process, as it happens in the pathogenesis of colorectal cancer. Unfortunately, an imaging classification of ADPKD patients to identify those at risk of rapid disease progression or malignancy transformation is not yet standardized; this may be very useful in selecting patients for clinical management.

As reported previously, nephrectomy for enlarged polycystic kidneys in patients with ADPKD is associated with significant complication rates [28]. The most common in our series was the necessity for transfusion, as confirmed by the haemoglobin drop postoperatively in both the transplant and dialysis groups. Literature confirms that avoiding simultaneous nephrectomy and transplantation reduces by approximately 40% the risk for postoperative complications and by 100% the risk for blood transfusion [29]. We therefore aim to operate on the patients awaiting kidney transplantation once they are established on dialysis. The only change in anti-HLA level post-native bilateral nephrectomy occurred in a female dialysis patient, with previous history of pregnancies but without being transfused. Eight patients were transplanted after bilateral native nephrectomy without complications.

A death incidence of up to 8.6% following nephrectomies of APKKD has been reported in literature [30]. There was one death in our cohort (3 %) for a patient who was initially discharged home but died six months later. This patient experienced refractory ascites eventually leading to hepatorenal syndrome (Clavien grade IV). This unfortunate outcome was not predictable on the basis of the liver function tests that were normal preoperatively. Patients with liver involvement require a combined liver–kidney transplantation only in cases of symptomatic hepatomegaly or recurrent cholangitis and if the glomerular filtration rate is ≤30 ml/min/1.73 m^2^ [21]. There is however significant concern in this population for the avoidance of poor postoperative outcomes [31] and there is no standardised practice for their treatment. An association with polycystic liver disease was present in 18 cases in our series (p<0.001), and the only patient who developed hepatic insufficiency had normal liver function tests, no previous history of liver symptoms, and also a working transplant at the time of the native bilateral nephrectomy and so theoretically was in a lower risk group. The other complications that occurred, i.e., ileus (Clavien grade II) or abdominal collection (Clavien grade II/III), did not significantly affect the LOS, and 32 of the 33 patients were well in the post-native bilateral nephrectomy follow-up.

## 5. Conclusion

In our experience, native bilateral nephrectomy for ADPKD can be performed safely and it is recommended for patients who need space for a future kidney transplant or have significant refractory symptoms or the suspicion of cancer. Furthermore, although this operation is associated with an increased risk of postoperative transfusion, it does not affect kidney graft function and does not pose an increased immunological risk. We recommend annual screening for the complications that may occur in this particular cohort of patients.

## Figures and Tables

**Table 1:** Results: the estimated Glomerular Filtration Rate (eGFR) is expressed in ml/min/1.73m^2^ and the haemoglobin (Hb) in g/L.

		Total (%)	Median (Range)

Male		18 (53)	

Age at operation (years) (both groups)			55 (27-71)

Age at operation (years) (transplant group)			54 (27-68)

Age at operation (years) (dialysis group)			59 (40-71)

Indication	Discomfort/space	20 (59)	
	Infection	12 (36)	
	Haematuria	5 (15)	
	Pain	8 (24)	
	Weight loss and cystic content modification	1 (3)	

Transplant pre-native bilateral nephrectomy		18 (53)	

Transplant post-bilateral native nephrectomy		8 (24)	

eGFR pre-nephrectomy (18 patients-transplant group)			44 (29-73)

eGFR day 1 post-nephrectomy (18 patients-transplant group)			45 (27-90)

Hb pre-native bilateral nephrectomy			121 (82-150)

Hb day 1 post-native bilateral nephrectomy			107 (58-154)

Transfusion		14 (42)	

Anti-HLA or DSA level change		1 (3)	

Length of hospital stay (days)			9 (6-71)

DSA: Donor Specific Antibody. HLA: Human Leukocyte Antigen.

**Table 2:** Incidental lesion findings in relation to nephrectomy indication and timing from transplantation.

Histology	Indication	Transplant pre-nephrectomy	Time from Transplant (months)	Follow-up (months)

4 mm papillary adenocarcinoma	Pain	Yes	156	17.7

1.5 mm papillary adenoma	Space	Yes	19	13.8

1 mm papillary adenoma	Space	No	/	6.7

1 mm papillary adenoma	Space, infection, haematuria, cystic content change	No	/	2.5

0.5 mm papillary adenoma	Space, infection	No	/	26.7

## Data Availability

The data used to support the findings of this study are included within the article.

## Abbreviations

DSA: Donor Specific Antibody
Hb: Haemoglobin
HLA: Human Leucocyte Antigen
LOS: Length of Hospital Stay
RCC: Renal Cell Carcinoma.
